# Food Sources of Potassium in the Average Polish Diet

**DOI:** 10.3390/nu11122905

**Published:** 2019-12-01

**Authors:** Hanna Górska-Warsewicz, Krystyna Rejman, Wacław Laskowski, Katarzyna Kowalcze

**Affiliations:** 1 Institute of Human Nutrition Sciences, Department of Food Market and Consumer Research, Warsaw University of Life Sciences, 02-787 Warsaw, Poland; krystyna_rejman@sggw.pl (K.R.); waclaw_laskowski@sggw.pl (W.L.); 2 Faculty of Medical Sciences and Health Sciences, Siedlce University of Natural Sciences and Humanities, 08-110 Siedlce, Poland; katarzyna.kowalcze@uph.edu.pl

**Keywords:** potassium intake, nutrient intake, food sources

## Abstract

The aim of this study was to identify the food sources of potassium in the average Polish diet based on the data from the 2016 Household Budget Survey conducted on the representative sample of the Polish population (36,886 households, *n* = 99,230). This survey is organized by the Central Statistical Office and is related to the expenditures, quantitative consumption and revenues in households. We analyzed 91 sub-groups (i.e., milk, red meat) from 13 food categories (i.e., milk and dairy products, meat and products). Our findings indicated that the daily supply of potassium in the average Polish diet was 2617.9 mg, which meant covering the average allowance in 83%. Vegetables provided 32.5% of potassium, of which potatoes accounted for 16.2% of supply, and other vegetables for 16.2%. Tomatoes as well as other vegetables and mushrooms provided a total of 8.2% of potassium among vegetables. The next position was taken by the meat and meat products category (17.7%), with the largest share of meat products (6.7%) and red meat (5.2%). Cereal products supplied 16.64% of potassium, of which bread, rolls and bread products (12.2%) were of the greatest importance. Milk and dairy products turned out to be the fourth product category as a source of potassium (11.9%), with the highest share of milk (6.8%) and yoghurts and milk drinks (3.9%).

## 1. Introduction

Potassium is a nutrient that influences many processes in the human body by performing various biological functions [1,2,3,4,5,6]. It is a cofactor involved in protein synthesis [1,6,7], activation of enzymes, participation in water balance and thus affecting osmosis [6]. It is required for insulin secretion, creatine phosphorylation and carbohydrate metabolism [1]. Adequate levels of potassium in the body have a positive effect on the vascular endothelium, and also reduce the production of free radicals, thereby reducing the risk of stroke [8].

Potassium is perceived as a “nutrient of public health concern” because people are not meeting their estimated recommended dietary intake [9]. Diets high in potassium are linked to reduction in blood pressure [4,7,9,10,11,12,13,14], decreasing the risk of stroke [9,10,15,16], improving bone health, and reducing the risk of nephrolithiasis [9,10]. WHO strongly recommends an increase of dietary potassium intake to reduce blood pressure and risk of cardiovascular disease, stroke and coronary heart disease in adults [17]. Today, the majority of people in the world consume a diet relatively high in salt (NaCl) and low in potassium (K+). A high dietary sodium (Na+) to K+ ratio is associated with hypertension, cardiovascular disease, and all-cause mortality [13,14]. Therefore, the recommendations and population studies highlight the role of health lifestyle, including adequate consumption of vegetables and fruits, which are a source of potassium [18].

Potassium is the major intracellular cation (with 98% of K+ located in the cells at a concentration of 140–150 mmol/L), and only 2% in the extracellular fluid (3.5–5 mmol/L) [19]. The consequence is that even small changes in potassium levels can be life—threatening [20]. This also determines the impact on the maintenance of normal cell functions [21]. The tissues most severely affected by potassium imbalance are muscle and renal tubular cells [7]

Disturbances of potassium homeostasis can cause hyperkalemia or hypokalemia and results in serious consequences [2,8,19,21,22,23]. They are related with the role of potassium in health and disease, focusing on cardiovascular, nutritional, and kidney considerations [2]. Hypokalemia and hyperkalemia are common electrolyte disorders caused by changes in potassium intake, altered excretion, or transcellular shifts. Diuretic use and gastrointestinal losses are common causes of hypokalemia, whereas kidney disease, hyperglycemia, and medication use are common causes of hyperkalemia. Severe, potassium disorders can lead to life-threatening cardiac conduction disturbances and neuromuscular dysfunction. These symptoms are: muscle weakness, impaired smooth muscle spasm (constipation intestinal paralysis), myocardial dysfunction (disorders rhythm), impaired renal function, paresthesia, hyperactivity, apathy, impaired concentration, cold intolerance, and many others [24,25,26]

Based on these arguments, the identification of the amount of potassium intake and its dietary sources is important in the context of public health. Therefore, the purpose of our research was to identify food sources of potassium based on the data from the 2016 Household Budget Survey in Poland. We analyzed the structure of particular food categories and product groups in total potassium supply and investigated the impact of socio-demographic and economic characteristics of the households on the volume and structure of potassium supply to the average diet.

## 2. Methods

### 2.1. Study Overview

We analyzed food sources of potassium, including 13 categories of food products, 42 main product groups, and 91 sub-product groups/food products. This is the next stage of our research relating to food sources of nutrients [27] and the role of individual product groups in the supply of energy, macronutrients, minerals, and vitamins [28,29,30]. The research process consists of 7 stages:

1. Two-stage random selection of representative sample of households conducted by the Central Statistical Office (Section 2.2);
2. recording purchase and consumption data per month in each household (Section 2.2);
3. conversion of consumption data into one person per month in each household (in g, kg, liters per person per month)—own calculation (Section 2.4);
4. conversion of consumption data into potassium content—own calculation (Section 2.4);
5. calculation of average potassium content in sub-groups in mg per person per day in all households (Section 2.4);
6. calculation of average potassium contribution (in %) to the average Polish diet from each sub-groups, main food groups and food categories—own calculation (Section 2.3, Section 2.4); and
7. cluster analysis of the impact of socio-demographic and economic factors on the level and structure of potassium supply—own analysis (Section 2.4).

### 2.2. Sample Selection Method

Household Budget Survey (HBS) is the representative survey conducted systematically by the Central Statistical Office (CSO) in Poland. The Social Surveys and Living Conditions Statistics Department of the CSO and the Statistic Office in Łódź deals with the cyclical organization of the survey, its conduction and control. The representative method is based on a random sample, which gives the opportunity to generalize the results to all households in Poland [31,32,33]. In 2016, 36,886 households (*n* = 99,230) participated in the study, and their random selection was two-stage. In the first stage, area survey points and—in the second one—flats and apartments were drawn. It was assumed that area survey points should include at least 250 apartments in the city, and 150 in the countryside. The basis for the draw in the second stage were lists of inhabitants prepared by statistical offices. To ensure representativeness—in addition to random sample selection—a random method of replacing households is used in the case of refusal to participate in the survey. For this purpose, the CSO uses two random lists of households (first and second selection) to achieve the planned sample of households [32].

In each household, expenditures, quantitative consumption and revenues are recorded in special budget books “Household Budget Diary” (paper booklet or electronic version) for one month. A questionnaire called “Household’s Statistical Sheet” is also completed [32,34]. Survey is carried out by employees of voivodship units of statistical offices. Their official duties include: Conducting training in the field of recording purchases and consumption, visiting each household 4 times a month, checking the collected and entered information, analyzing the recorded data, explaining inaccuracies and entering the data into the CSO system. In addition, an interview is conducted in every household [34].

### 2.3. Food Grouping

The HBS includes 91 food sub-groups/food products (Table 1). We made a classification for the needs of our analyzes [27,28,29,30] based on previous classifications [35,36,37,38] and the specificity of products available on the Polish market [34].

### 2.4. Data Analysis

Data on the volume of purchase and consumption of 91 sub-groups/food products per one month in each household was converted per one person. For each of 91 sub-groups we have received the amount of consumption in grams, kilograms, or liters per one person per one month and one day. The next step was the conversion of consumed amount of individual food products into energy, macronutrients, minerals and vitamins. For the purposes of our study, we have considered potassium. For calculations, we used the current version of nutritional value tables “Nutritive Value Tables for Foods and Meals” (4th ed.) [39], we made calculations using the R software environment for statistical computing (v 3.0.2) [40,41,42]. To improve the representativeness of the results, we used the tools available in this system [40,42]. This allowed generalizing the results obtained to the population of the whole country [43,44]. More detailed descriptions and calculation schemes have been published in our earlier articles [27,28,30].

In addition, potassium supply was compared to the reference value. For this purpose, we used the reference value for potassium for the Polish population calculated for our sample of households. The basis of calculations were the references developed and published by the National Food and Nutrition Institute [45].

To analyze the impact of socio-demographic and economic factors on the potassium supply in the average Polish diet, exploratory data analysis (EDA) was applied [46,47,48]. In our calculation 14 factors were included to divide the analyzed sample of households into 3 clusters differing in the amount of potassium supply. This parameter, i.e., the daily supply of potassium, was the basis for the cluster analysis carried out using the Kohonen Neural Network [49]. The description of clusters include: Number of people in household, family life phase, age, income (quintile group), socio-economic affiliation, month of study, sex, region, education level, land use, assessment of financial situation, assessment of nutrition, size of the village, and degree of urbanization of place of household living. For each factor, a correlation table was created together with a chi^2^ test and a measure of Cramer’s correlation.

## 3. Results

### 3.1. Sample Characteristics

In 2016, 36,886 households were randomly drawn for the HBS survey. The total number of members of these households was 99,230. Detailed socio-demographic and economic characteristics of this sample are presented in Table 2.

### 3.2. Food Sources of Potassium—General Overview

The daily supply of potassium in the average diet was 2617.9 mg (Table 3), of which vegetables, meat and its products as well as cereal products provided 2/3 of the daily supply of this nutrient. Also including—alongside these three product categories—milk and dairy products—gives a share in the daily supply above ¾.

### 3.3. Food Sources of Potassium—Detailed Analysis

#### 3.3.1. Vegetables as Sources of Potassium

There are two equal groups in the structure of vegetable supply: Vegetables (excluding potatoes) and potatoes (Table 4). Sub-groups of other vegetables and mushrooms (5.1%), tomatoes (3.1%), and processed food products from vegetables and other mushrooms (2.0%) have the largest share in the structure of vegetable supply. Other vegetables include peppers, kohlrabi, zucchini, green peas with pods, sweet corn, other root and tuber vegetables (e.g., radish, celery, leek, asparagus), pulses, including peas, beans, lentils, and soybean. Vegetable and mushroom preserves, in turn, include dried vegetables and mushroom, pickled and salted vegetables, pickles, concentrates, purees, salads, mushroom preserves, dietary foods the main ingredient of which are vegetables.

In the group of potatoes and potato products, potatoes with a share of 15.1% in potassium supply and potato products (industrially manufactured), including puree, potato flakes, fries, and potato dumplings, were distinguished due to the specificity of the Polish diet.

#### 3.3.2. Meat and Meat Products as Sources of Potassium

The category of meat and meat products provided 17.7% of potassium in the daily supply of this nutrient (Table 5). The largest share in the supply structure was represented by meat products (6.7%), in particular processed red meat products (4.9%). Next positions were occupied by red meat (5.2%), mainly pork (4.8%) and poultry (5.0%), mainly chicken (4.3%).

#### 3.3.3. Cereal Products as Sources of Potassium

Cereal products provided 16.6% of the daily potassium supply to the average Polish diet (Table 6). Bread, rolls, bread products (12.2%) had the largest share, including bread and rolls (10.1%). Quick breads, bread products were responsible for providing 2.1% of potassium.

#### 3.3.4. Milk and Dairy Products as Sources of Potassium

The share of milk and dairy products in the average supply of potassium was 11.9% (Table 7). The highest percentage came from milk (6.8%) isolated as main food group, predominantly whole milk (4.1%). Yogurts and milk drinks with the share of 3.9% came second, including milk shakes and other dairy drinks i.e., kefir, buttermilk, flavored milk (2.3%), and yoghurts (1.6%).

#### 3.3.5. Fruits as Sources of Potassium

The share of fruit, dried fruits and nuts in the structure of potassium supply was 8.6%, of which 7.6% was fruits (Table 8). In the structure of fruit consumption, the largest share in potassium supply was found in bananas (2.1%), followed by apples (1.4%), citrus fruits (1.3%), and berries (1.0%). The share of other fruits was below 1%.

### 3.4. Potassium Intake Levels by Socio-Demographic and Economic Characteristics of the Survey Population

To analyze the impact of socio-demographic and economic factors on the volume and structure of potassium supply to the average diet, a cluster analysis was conducted. Factors that had the greatest impact were: Number of people in household, family life phase, age, income (in quintile group), and socio-economic affiliation (Table 9).

Three clusters with different levels of potassium supply were identified: Below 2000 mg of potassium per day, 2000–3000 mg and above 3000 mg (Table 10). Cluster no. 3 (with average potassium supply above 3000 mg) had the highest share of vegetables, including potatoes in the supply of potassium (38.7%), while in Cluster no. 1 with the lowest potassium supply (below 2000 mg) we observed the smallest share of vegetables in potassium supply (26.8%) was observed. For factors with the greatest impact on the volume and structure of potassium supply, we also presented the characteristics of clusters (Table 11).

## 4. Discussion

The aim of our study was to identify the food sources of potassium in the average Polish diet based on the 2016 HBS data. We analyzed 13 food categories, 42 main product groups and 91 sub-product groups/food products. The obtained results were compared with other results published in the scientific literature related to the Dutch [50], French [51], American [36,52,53], New Zealand [54], Brazilian [55], Taiwanese [56], and Australian [57] diets.

The daily supply of potassium in the average Polish diet amounted to 2617.9 mg, which meant covering the average allowance for the population (at a level of adequate intake) in 83%. WHO suggests an intake of at least 90 mmol/day (3510 mg/day) for adults [17], as an adequate supply of potassium as well as calcium and magnesium is crucial for the prevention of hypertension [58]. The supply of potassium in the Polish diet corresponds to 75% of this recommended amount. In this context, it should be noted that cardiovascular diseases were the cause of 43% of deaths in Poland in 2016, which was the highest percentage of all causes of death. In this number, coronary heart disease accounted for almost a quarter [33]. Insufficient potassium intake was also found in the prospective cohort ‘PONS’ study conducted in one of the least developed regions of Poland in 2011 (3862 adults, FFQ method). Potassium intake was calculated at 2453 mg/day in men and 2497 mg/day in women [59]. A study on a group of postmenopausal women in Poland found that most of them had insufficient potassium intake (406 women, method of dietary record, conducted during two typical, non-consecutive days) [60]. In the National Multicenter Health Survey (WOBASZ II, random sample of 5690 adults aged 20 years and above, 24-h dietary recall method), conducted in Poland in 2013–2014, potassium intake was 3467 mg/day in male participants and 2862 mg/day in female participants, so only 17% of male and 5% of female subjects met the recommendations [58].

The level of potassium intake in the Polish households reflects the observation of Cohn et al. (2000), as according to their study urban whites typically consume approximately 2500 mg (62.5 mEq) of potassium daily [7]. EFSA’s analysis based on the data from 13 dietary surveys in 9 EU Member States (2000 and 2011 studies) showed that the potassium intake by age groups of population. The average daily intake of potassium in infants ranged from 821 to 1535 mg, 1516–2005 mg in children aged 1–3 years, 1668–2750 mg in children aged 3–10 years, 2093–3712 mg in teenagers aged 10–18 years and 2463–3991 mg in adults [61]. Studies on the Brazilian population also indicate lower potassium intake compared to the average allowances [55].

In the US, potassium is among three nutrients of public health concern [36]. On the basis of 2011–2012 National Health and Nutrition Examination Survey (NHANES) data, US adults consumed daily 2800 mg potassium on average and the intake was significantly lower for blacks than for all other racial groups, overall and among women. The analysis revealed that level of potassium intake represents only 60% of the average allowance for US adults and only about 3% of them meet the recommendations [52]. According to data from the 2015–2016 NHANES, the average daily potassium intake from foods was 2227 mg for males aged 2–19, and 1943 mg for females aged 2–19 [53]. In adults aged 20 and above, the average daily potassium intake from foods was 2967 mg for men, 2323 mg for women, and 2633 mg for all adults. This represents a decrease of 5% in potassium intake compared to the results of 2011–2012 NHANES. In the entire US population, the average intake of potassium was 2502 mg, which is 4% less than our calculations for the Polish population. Potassium is present in a wide variety of natural foods, of plant and animal origin, and in coffee, tea, and other nonalcoholic beverages. In particular, starchy roots or tubers, many vegetables, pulses, and fruits are excellent sources of potassium [36,62]. In the average Polish diet vegetables and potatoes were the main food category supplying potassium, contributing 32.5% of its daily intake. In this category vegetables provided 16.2% of potassium, of which sub-group of other vegetables and mushrooms was the main supplier contributing 5.2%, followed by tomatoes—3.1%. Data from the analysis of the average American adults’ (19+ years) diet indicated the share of tomatoes and tomato/vegetable juice at the level of 5.9% in potassium supply, other vegetables—3.4% and pulses—2.6% [36], which gives a total of 11.9% share of the vegetable group. Research conducted in the Dutch, New Zealand, and French populations indicated the total share of vegetables in potassium supply—at 9% [50], 11.6% [54], and 19.5% [51] respectively. In the average Australian diet, the share of vegetables, vegetable products and dishes excluding potatoes in potassium supply was 10.6%–13.1% (women-men), including tomatoes and tomato products 2%–2.4%, cabbage, cauliflower and similar brassica vegetables 1.9%–2.3%, carrot and similar root vegetables 1.4%–1.6%, leaf and stalk vegetables 1.1%–1.5%, other fruiting vegetables 1.9%–2.7%, and other vegetables and vegetable combinations 1.3%–1.5% [57].

Our research has shown that potatoes and their products (industrially manufactured) delivered almost the same amount of potassium as the vegetable sub-group, as they contributed 16.2% of this nutrient to the average Polish diet, of which potatoes accounted for 15.1% of the supply. In the average American diet, the share of potatoes was 6.7% [36], in Australia—12.4%–13.8% [57], and in Dutch population the share of potatoes and other tubers in the average diet was at the level of 11% [50]. Similar results were obtained for potatoes, kumara and taro in the average New Zealand diet—12.7% [54]

The consumption of potatoes in Poland is constantly decreasing, which means that it plays a lesser role in the supply of energy and all the nutrients. In 2006, household consumption of potatoes amounted to 68.6 kg [63], while in the analyzed year (2016) it was lower by 39% and amounted to 41.8 kg [31]. In the same period, the consumption of vegetables was slightly reduced, by 4%. Nevertheless, potatoes as a food sub-group are still the main source of potassium in the average Polish diet, providing 15% of this nutrient in the diet. People who eat large amounts of fruits and vegetables tend to have a high potassium intake of approximately 8000 to 11,000 mg/d (200–250 mEq) [7]. It seems that this level of potassium intake from food can be achieved by people on a vegetarian diet, because current dietary patterns in highly developed countries are characterized by insufficient consumption of fruit and vegetables, below 400 g per day, which is recommended by FAO and WHO experts [64]. This is indirectly evidenced by the above mentioned data on potassium deficiency in the populations of various countries. In the average Polish diet, vegetables (including potatoes) and fruit together provide 41.1% of potassium, while in the diet of adult Americans—29.4% [36].

Meats, poultry, fish, milk, yogurt, and nuts also contain significant amount of potassium [62]. The share of meat and meat products in the potassium supply in the average Polish diet was 17.7%, of which meat products supplied 6.7% potassium, red meat 5.2%, and poultry 5%. In the average American diet, the share of meat products in the supply of potassium was at the following level: Beef—5.2%, poultry—3.9%, pork, ham, and bacon—2.9%, and frankfurters, sausages, and luncheon meats—2.2% [36], which gives 14.2% share of this group in the supply of potassium in the diet. The following data was obtained for the average New Zealand diet: Beef and veal 3.6%, poultry 3.5%, pork 2.5%, and sausages and processed meats 1.2% [54], which adds up to 10.8%. In Dutch and French population, meat and meat products contributed 13% [50] and 11.3% [51] to the average potassium intake, respectively. Similar results were obtained in Australia. The share of meat, poultry and their products and dishes in the potassium supply was 11.9%–15.5%, including muscle meat 3.8%–5.6%, poultry 1.3%–1.5%, and mixed dishes (where beef or veal is the major component) 3.6%–4.4%, mixed dishes where poultry is the major food type 1.6%–1.8% [57].

Our research indicated that cereals and cereal products as a food category were the third source of potassium in the average Polish diet, providing 16.6% of this nutrient. Bread, rolls, and bread products had the highest share (12.9%), and in this group, bread and rolls were the main contributors (10.1%). In the average American diet, yeast breads and rolls provided 2.8% of potassium and cakes, cookies, quick bread, pastry, pie 2.1% [36]. In New Zealand, bread accounted for 5.5% of potassium supply, grains and pasta—for 3.9%, bread-based dishes for 3.7%, and breakfast cereals for 2.2% [54], which gives 15.3% share of this food group in the supply of potassium in the diet. In the Dutch diet, cereal products provided a total of 12% of potassium [50]. For the Australian diet, the share of cereal products in the potassium supply was 9.2%, including regular breads, and rolls 4.3%–4.5%, and breakfast cereals, and mixed source 1.6%–1.7% [57].

Milk and dairy products are an important source of potassium [65,66,67] and this food category placed as fourth source of this nutrient in the average Polish diet. The share of milk and dairy products in the average diet was 11.9%, including 6.8% from milk and 3.9% from yoghurts and milk drinks. The share of milk in the potassium supply in the average American diet was 9.6% [36], in the Dutch diet 17% [50] and among French population 11.3% [51]. In the New Zealand diet, milk provided 9.8% potassium, and dairy products 2.3% [54]. Milk and dairy food category has a slightly higher share of 14.1%–15.5% potassium supply in the Australian diet, including milk 9.7%–11.0%, yoghurt 0.7%–1.3%, frozen milk products 0.9%–1.2%, and flavored milk 0.7–1.3% [57]. However, in the average Taiwanese diet, milk and dairy products were the one of the most important source of potassium, accounting for 15.7% of the average daily supply [56].

The cluster analysis, in which we distinguished three clusters, showed that the volume and structure of potassium supply depend on the characteristics of the surveyed households. Consumption of potassium in the Cluster 1 was lower than 2000 mg, in the Cluster 3 it was at least 50% higher. In the structure of potassium supply we noted two important features. Firstly, the share of 5 food categories (out of 9 analyzed) was decreasing from the first to the third Cluster. These were: meat and meat products (in Cluster 3 the share was lower by 15% compared to Cluster 1), cereal products (by 26%), milk and dairy products (by 19%), nonalcoholic beverages (by 28%), and snacks, sweets and sugars (by 26%). Secondly, the share of 2 food categories was increasing from the first to the third cluster, i.e., of vegetables and fruits. The share of vegetables in the Cluster 3 was higher by 44% higher, and fruit by 19%. Taking this into account, it can be said that the diet of people in the Cluster 1 was the least advantageous in terms of health. Households in this Cluster distinguished the following socio-demographic and economic characteristics:

- The largest number of households with 3, 4 or more persons (70.5% in Cluster structure), and the share of multi-person households (5 or more) was over 5 times higher than in the Cluster 3;
- almost half of them were families with children at pre-school and school age (48.3%), while in the Cluster 3 the highest share of singles’ and young marriages households (15.8%) and older marriages (55.2%) was recorded;
- these were households of young people (up to less than 40 years old—35.8%, less than 50 years old—59.0%); in the Cluster 3 people aged 50 and more dominated (70.1%);
- more than half of these households had low income (54.9% in the first and second quintile group), while in the Cluster 3 half of households had high income (50.8% in the fourth and fifth group); and
- almost 1/3 of these households were households of blue-collar workers; at the same time in Cluster 1 the largest share of self-employed households (8.4%) and those in a very difficult income situation (living on social benefits or from other unearned sources) were recorded; in the Cluster 3 the share of pensioners and pensioners was the largest (46.5%).

On the basis of cluster analysis, it can be concluded that nutrition education programs should be addressed to multi-person households, young people, with dependent children, with low incomes, and blue collar workers. It should be stressed that as early as in 2000, in most population groups in the households (in terms of gender and age), the daily intake of potassium corresponded to the level specified in the dietary allowances [68].

According to the WHO strong recommendation, an increase in potassium intake from food is needed to reduce blood pressure and the risk of cardiovascular disease, stroke and coronary heart disease. WHO experts suggest for adults a potassium intake of at least 90 mmol/day (3510 mg/day) for adults. They suggest also an increase in potassium intake from food to control blood pressure in children. The level of potassium intake recommended for adults should be adjusted downward for children based on their energy requirements. However, the dietary patterns of many populations are characterized by too low intake of potassium and much higher than the recommended an intake of sodium. Therefore, the dietary guidelines should be updated with regard to the need to increase the intake of potassium. This postulate should be addressed to policy-makers in the interest of improving public health and reducing health-care costs. This is another argument in favor of an increase in plant consumption, since plants are the main source of potassium in the diet. The WHO gives the examples of food products with high potassium content: different beans and peas, nuts, green vegetables, root vegetables (carrot, onions, beetroot), other vegetables (tomatoes, cucumbers, pumpkins), and fruits (bananas, papayas, and dates) [17]. Therefore, we conducted calculations based on a representative sample of the Polish population to see what the main food sources of potassium in the average Polish diet are. Insufficient potassium supply in the current Polish diet occurred due to the unfavorable changes in food consumption patterns. The transition to a sustainable diet is now highly desirable. Such a diet contains more pulses and nuts, which are a very good source of potassium and will increase it intake. To implement this diet, numerous and varied educational measures are needed to convince people to increase their consumption of vegetables, including pulses, nuts and to stimulate renewed interest in eating boiled potatoes and potato dishes. Our research has shown which groups of households should be targeted first and foremost. It should be noted that these actions will require support (also financial) from the government and public institutions as a part of the food and nutrition policy.

## 5. Conclusions

Our results showed that people in Poland consume on average less potassium than recommended. There are significant differences in the population with regard to the amount of potassium intake and the role of particular food categories in its supply. Number of people in a household, family life phase, age, income (in quintile group), and socio-economic type of household determined the occurrence of these differences to the greatest extent.

In the Polish population, potassium deficiency deepen, as a consequence of changes in food consumption patterns occurring with an increase in the standard of living of the population. Our results, covering a representative group of households from all over the country, confirm the results of other studies conducted with different methods and on smaller and different population groups. An analysis based on the HBS data has allowed us to take into account all-year-round food consumption and thus identify potassium sources in the average diet. Such information was not provided by other studies on potassium intake in the Polish population. In our study, we have identified four food categories as the main sources of potassium in the average Polish diet, which together account for almost 79% of this nutrient. Vegetables, including potatoes, provided 32% of potassium in equal amounts. The next sources of potassium, supplying more than 10%, were meat and meat products (18% of the total amount of potassium in the diet), cereal products (17%), milk and dairy (12%). As fruit consumption in Poland still remains low (on average 44 kg per person in the household in 2016), the contribution of this food category in potassium supplying equals less than 9%. It should be noted, however, that our calculations are based on the household food consumption and therefore do not include eating out of home. However, Polish households spend relatively little on eating out of home. In 2016, this was 16% of total expenditure on food and non-alcoholic beverages. The second limitation of our results is that potassium is a component of wide range of dietary supplements. Also, many salt substitutes contain potassium chloride as a replacement for some or all of the sodium chloride in salt. The results of our analysis may be helpful in developing dietary guidelines and educational programs for special population groups and in the practice of dietary guidance in order to achieve better health status both of the population and individuals.

## Figures and Tables

**Table 1:** Food grouping for the purpose of food sources analysis.

Food Categories	Main Food Groups	Sub-Groups/Food Products
GRAIN PRODUCTS	bread, rolls, bread products	(1) bread and rolls (2) quick breads and bread products
	rice, cooked grains	(3) rice (4) groats (5) cereal grains
	flour, bran, cooking ingredients	(6) wheat flour (7) other flours
	pizza, pasta, and other flour dishes	(8) pasta (9) macaroni, noodle (10) pizza (11) other flour dishes
	ready-to-eat cereal	(12) breakfast cereals
MEAT AND MEAT PRODUCTS	red meat	(1) beef (2) pork (3) sheep, goat (4) veal
	meat products	(5) processed red meat products (6) processed poultry products (7) other meat products
	other meat	(8) liver organ meat (9) minced meat (10) other meat
	poultry	(11) chicken (12) other poultry
MILK AND DAIRY PRODUCTS	milk	(1) milk, whole (2) reduced fat milk (3) condensed and powdered milk
	cheese	(4) cheeses
	cottage cheese	(5) cottage cheeses
	yoghurts and milk drinks	(6) yogurts (7) milk shakes and other dairy drinks
SEAFOOD	fish	(1) fresh, chilled or frozen fish
	shellfish	(2) fresh, chilled or frozen shellfish
	processed seafood	(3) dried, smoked and salted seafood (4) other fish and shellfish products
EGGS	eggs	(1) eggs
FATS AND OILS	butter	(1) butter
	olive oils other oils	(2) olive oil (3) other oils
	other fats	(4) margarine and other plant fats (5) other animal fats
	sour cream	(6) cream
FRUITS	fruits	(1) apples (2) bananas (3)berries (4) citrus fruits (5) frozen fruits (6) fruits products (7) other fruits (8) peaches and nectarines
	dried fruits and nuts	(9) dried fruits and nuts
VEGETABLES	potatoes	(1) potatoes (2) potato products
	vegetables (excluding potatoes)	(3) beetroot (4) cabbage (5) carrot (6) cauliflower (7) cucumber (8) lettuce (9) onions (10) tomatoes (11) frozen vegetables and mushrooms (12) sour cabbage (13) other vegetables and mushrooms (14) vegetable and mushroom products
SNACKS AND SWEETS	chocolate	(1) chocolate (2) powdered cacao (3) powdered chocolate
	desserts	(4) ice-cream
	snacks	(5) chips
	sweet bakery products	(6) cakes and pies
SUGARS	honey	(1) honey
	jams, syrups, marmalade	(2) jams (3) syrups (4) marmalade
	sugar	(5) sugar
	sugar substitutes	(6) sugar substitutes
BEVERAGES, NONALCOHOLIC	juices	(1) fruit juices (2) vegetables juices (3) mixed juices
	other beverages	(4) other nonalcoholic beverages
	water	(5) water
COFFEE, TEA	coffee	(1) coffee
	tea	(2) tea
ALCOHOLIC BEVERAGES	wine	(1) wine (2) wine-based beverages
	beer	(3) beer, lager (4) low-alcohol and non-alcohol beer (5) beer-based beverages
	other alcoholic beverages	(6) liquor and cocktail (7) other alcoholic beverages

**Table 2:** Sample characteristics.

Specification	Structure in %
**Sex**	
women	52.4
men	47.7
**Age**	
18– >30 years	7.6
30– > 40 years	17.5
40– >50 years	17.8
50– >60 years	19.5
60– >70 years	21.3
**Number of People in Household**	
1	20.6
2	32.8
3	19.8
4	16.6
5 and above	10.3
**Education Level**	
higher	18.9
secondary vocational or post-secondary	21.8
secondary general	9.6
basic vocational	27.4
lower secondary	5.1
primary	17.5
**Family Life Phase**	
Singles, young marriages	13.9
Families with preschool children	15.6
Families with school children	14.4
Families with trainees	15.0
People, older marriages, professionally active	20.4
People, older marriages, professionally inactive	20.8
**Income (in Quintile Group)**	
1 (20% of people with the lowest income)	20.0
2	20.0
3	20.0
4	20.0
5 (20% of people with the highest income)	20.0
**Socio-Economic Group**	
employees in worker positions	24.5
employees in non-worker positions	24.0
farmers	4.6
self-employed	6.8
pensioners	29.9
disability pensioners	6.3
living on social benefits	2.6
living on other unearned sources	1.5
**Assessment of Own Financial Situation**	
good	13.1
rather good	19.8
average, neither good nor bad	54.3
rather bad	9.5
bad	3.4
**Assessment of Nutrition in Household**	
good	46.7
rather good	28.4
average, neither good nor bad	22.8
rather bad	1.6
bad	0.5

**Table 3:** Potassium supply and sources of potassium contribution from food categories to the average Polish diet (in % of total potassium contribution).

Specification	Potassium
**Average daily intake of potassium in mg**	**2617.93**
**Average allowance (AI) in mg**	**3152.36**
**Fulfillment of reference value**	**83.05%**
**Structure of Potassium Intake in %:**	
Vegetables	32.47
Meat and meat products	17.69
Cereal products	16.63
Milk and dairy products	11.87
Fruits	8.59
Nonalcoholic beverages	4.45
Snacks, sweets ad sugars	4.21
Seafood	1.39
Eggs	1.16

Categories with a share of more than 1% in potassium supply are shown. The aggregate data are bold.

**Table 4:** Sources of potassium contribution from vegetables to the average Polish diet (in % of total potassium contribution).

Specification	Share of Potassium Intake
**VEGETABLES**	**32.47**
**Potatoes and Potato Products**	**16.23**
potatoes	15.07
potato products	1.16
**Vegetables (Excluding Potatoes)**	**16.24**
other vegetables and mushrooms	5.15
tomatoes	3.05
vegetable and mushroom products	1.99
carrot	1.36
cabbage	1.22
beetroot	0.66
cucumber	0.59
onions	0.58
frozen vegetables and mushrooms	0.57
sour cabbage	0.47
cauliflower	0.31
lettuce	0.26

Products providing more than 0.1% are included. The aggregate data are bold.

**Table 5:** Sources of potassium contribution from meat and meat products to the average Polish diet (in % of total potassium contribution).

Specification	Share of Potassium Intake
**MEAT AND MEAT PRODUCTS**	**17.69**
**Meat Products**	**6.68**
processed red meat products	4.88
other meat products	0.95
processed poultry products	0.85
**Other Meat**	**0.81**
liver and other offal	0.61
minced meat	0.15
**Poultry**	**5.03**
chicken	4.27
other poultry	0.76
**Red Meat**	**5.17**
pork	4.78
beef	0.37

Products providing more than 0.1% are included. The aggregate data are bold.

**Table 6:** Sources of potassium contribution from cereal products to the average Polish diet (in % of total potassium contribution).

Specification	Share of Potassium Intake
**CEREAL PRODUCTS**	**16.64**
**bread, rolls, bread products**	**12.19**
bread and rolls	10.11
quick breads, bread products	2.08
**flour, bran, cooking ingredients**	**1.35**
wheat flour	1.31
**pizza, pasta, macaroni and other flour dishes**	**1.37**
pasta, macaroni, noodle	1.01
pizza and other flour dishes	0.36
**ready-to-eat cereal**	**0.85**
breakfast cereals	0.85
**rice, cooked grains**	**0.87**
groats and cereal grains	0.53
rice	0.35

Products providing more than 0.1% are included. The aggregate data are bold.

**Table 7:** Sources of potassium contribution from milk and dairy products to the average Polish diet (in % of total potassium contribution).

Specification	Share of Potassium Intake
**MILK AND DAIRY PRODUCTS**	**11.87**
**cheese**	**1.21**
cheeses	0.57
cottage cheese	0.64
**milk**	**6.81**
condensed and powdered milk	0.23
milk, reduced fat	2.47
milk, whole	4.11
**yoghurts and milk drinks**	**3.85**
milk shakes and other dairy drinks	2.30
yogurts	1.55

Products providing above 0.1% included. The aggregate data are bold.

**Table 8:** Sources of potassium contribution from fruits to the average Polish diet (in % of total potassium contribution).

Specification	Share of Potassium Intake
**FRUITS**	**8.59**
**Dried Fruits and Nuts**	**0.99**
dried fruits and nuts	0.99
**Fruits**	**7.60**
bananas	2.13
apples	1.43
citrus fruits	1.28
berries	1.01
peaches and nectarines	0.84
other fruits	0.77
fruits products	0.11

Products providing more than 0.1% are included. The aggregate data are bold.

**Table 9:** Cluster analysis: Impact of socio-demographic and economic factors on potassium contribution to the average Polish diet.

Factors	Cramer Correlations
number of people in household	0.284
family life phase	0.224
age	0.175
income (quintile group)	0.170
socio-economic affiliation	0.145
month of study	0.098
sex	0.088
region	0.053
education level	0.041
land use	0.028
assessment of financial situation	0.028
assessment of nutrition	0.020
size of the village	0.016
degree of urbanization of place of household living	0.015

**Table 10:** Cluster analysis: level of potassium contribution by clusters.

Specification	Cluster 1	Cluster 2	Cluster 3	Whole Sample
Number of households	9406	12,947	14,533	36,886
Potassium contribution	below 2000 mg	2000–3000 mg	above 3000 mg	2617.93 mg
**Contribution of Potassium in % by Clusters From**				
Vegetables	26.8	33.0	38.7	32.5
(including potatoes)	10.9	15.8	19.4	15.1
Meat and meat products	19.0	17.6	16.2	17.7
Cereal products	19.0	16.4	14.0	16.7
Milk and dairy products	13.0	11.9	10.5	11.9
Fruits	8.0	8.4	9.5	8.6
Nonalcoholic beverages	5.1	4.4	3.7	4.5
Snacks, sweets ad sugars	4.7	4.2	3.5	4.2
Seafood	1.4	1.4	1.4	1.4
Eggs	1.2	1.2	1.1	1.2

**Table 11:** Cluster analysis: structure of clusters by 5 factors with highest Cramer Correlations.

Specification	Cluster 1	Cluster 2	Cluster 3	Whole Sample
**Whole Sample of Households**	25.5	35.1	39.4	100.0
**Number of People in Household**				
1	7.6	14.7	34.2	20.6
2	21.9	33.0	39.6	32.8
3	23.6	22.6	14.9	19.8
4	27.2	19.1	7.5	16.6
5 and above	19.7	10.6	3.8	10.3
**Family Life Phase**				
Singles, young marriages	11.3	13.6	15.8	13.9
Families with preschool children	25.8	17.2	7.5	15.6
Families with school children	22.5	16.3	7.4	14.4
Families with adult children	15.9	15.1	14.2	15.0
People, older marriages, professionally active	13.0	18.8	26.7	20.4
People, older marriages, professionally inactive	11.5	19.0	28.5	20.8
**Age**				
18 – >30 years	10.0	7.8	5.9	7.6
30 – >40 years	25.8	18.9	11.0	17.5
40 – >50 years	23.2	19.1	13.0	17.8
50 – >60 years	17.2	19.3	21.2	19.5
60 – >70 years	13.0	19.5	28.4	21.3
70 years and older	10.7	15.5	20.5	16.2
**Income (in Quintile Group)**				
1 (20% of people with the lowest income)	30.8	20.9	12.2	20.0
2	24.1	21.1	16.4	20.0
3	18.5	20.4	20.6	20.0
4	15.0	19.7	23.5	20.0
5 (20% of people with the highest income)	11.7	17.9	27.3	20.0
**Socio-Economic Group**				
employees in worker positions	32.3	25.7	18.4	24.5
employees in non-worker positions	27.1	25.2	20.9	24.0
farmers	3.6	5.0	4.9	4.6
self-employed	8.4	6.9	5.6	6.8
pensioners	19.1	28.1	38.4	29.9
disability pensioners	4.6	5.4	8.1	6.3
living on social benefits	3.3	2.5	2.2	2.6
living from other unearned sources	1.7	1.3	1.6	1.5
